# Supplementary material for: Development of Self-Administered Formulation to Improve the Bioavailability of Leuprorelin Acetate
Source: Pharmaceutics. 2022 Apr 3;14(4):785. doi: 10.3390/pharmaceutics14040785 (PMC9031317; doi:10.3390/pharmaceutics14040785)
Supplement: Supplementary file 1 [file pharmaceutics-14-00785-s001.zip › pharmaceutics-1623568-supplementary.pdf]

pharmaceutics

Supplementary Materials: Development of Self-Administered Formulation to Improve the Bioavailability of Leuprorelin Acetate

Akie Okada [1], Rina Niki, Yutaka Inoue, Junki Tomita, Hiroaki Todo, Shoko Itakura and Kenji Sugibayashi

【Method】

Liposomes composed of DOPG and DOPC with different molar mixing ratios (100:0, 75:25, 55:45, 25:75) were prepared with a thin-film method. The zeta-potential, particle size, and polydispersity index were measured by a Zetasizer Nano ZS (Mlavern Panalytical Ltd, Worcestershire, UK). A suitable liposomal composition in the present study was determined by the obtained parameters.

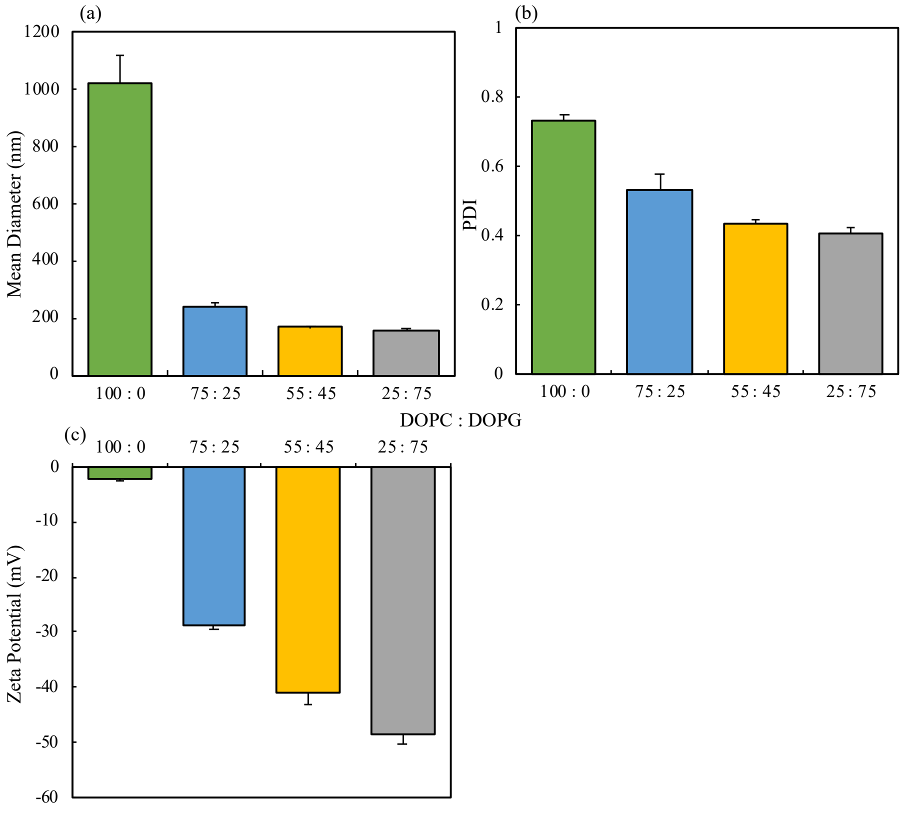

Figure S1. Effect of mixing ratio of DOPC and DOPG on the prepared particles of mean particle size, polydispersity index, and zeta potential.

The mixing ratio of DOPC:DOPG (75:25 molar ratio) was exhibited higher negative zeta potential with smaller particle size and a smaller polydispersity index value.
